# Molecular Mechanisms of Cardiac Development and Disease

**DOI:** 10.3390/ijms24108784

**Published:** 2023-05-15

**Authors:** Nicole Wagner, Kay-Dietrich Wagner

**Affiliations:** CNRS, INSERM, iBV, Université Côte d’Azur, 06107 Nice, France

During development, the heart is the first organ to form and function. Its work assures proper vital functions of the whole body throughout life. Cardiovascular diseases represent the leading cause of death worldwide. Spatial and temporal tightly controlled biological and molecular events happen during cardiac development and disease. Often, cardiac repair mechanisms in cardiovascular disease reflect developmental processes, involving the same cell types, transcriptional regulation processes, and molecular pathways. This highlights the importance of a profound understanding of the molecular mechanisms and different cell types involved in cardiac development to propose efficient therapeutic translation to clinical applications for the treatment of cardiovascular diseases. The present Special Issue of *International Journal of Molecular Science* aims to contribute to the knowledge of the molecular mechanisms of cardiac development and disease. It presents the most recent advances in understanding of the numerous aspects of the molecular regulation of cardiac development and disease, from basic science to applied diagnostic, therapeutic, and functional approaches, and provides new insights into the complex regulation of cardiac development, disease, and regeneration.

In this Special Issue, Fernando Bonet et al. describe the importance of the secreted extracellular matrix collagen- and calcium-binding EGF-like domains 1 (CCBE1) protein for epicardial function during cardiac development [1]. The epicardium, the outermost tissue layer that envelops all vertebrate hearts, plays a crucial role in cardiac development and regeneration and has been implicated in potential strategies for cardiac repair. Epicardial Epithelial-to -Mesenchymal Transition (EMT) is critical for providing support cells that contribute to myocardial integrity and involves several transcription factors (TFs) such as Wilms Tumor-1 Suppressor (WT1) (reviewed in [2,3,4]), the class II basic helix-loop-helix TF Tcf21, and serum response factor (SRF), among others (reviewed in [5]). Fernando Bonet et al. suggest that CCB1, known to be implicated in lymphangiogenesis, might additionally contribute to epicardial function. In vivo knockout of CCB1 reduced epicardial cell proliferation and migration, resulting in a diminished ventricular myocardium thickness. Epicardial CCB1 therefore seems to be implicated in the complexity of signals required for proper heart development [1]. The group around Francesca Boccafoschi explains the importance of focal adhesions (FAs) and Vinculin, the adaptor protein of FAs, for cardiomyocyte differentiation. FAs are a complex of proteins that localizes to sites of cell–matrix interaction and enables cell motility, migration, tissue organization and cytoskeletal dynamics. The authors demonstrate higher Vinculin expression and translocation during cardiomyocyte differentiation and mechanical stress application, resulting in reenforcing the integrin-based ECM adhesion complex. Taken together, their work underlines the implication of FAs for assurance of cardiac motility [6]. Shogo Hamaguchi and co-workers investigated in this Special Issue the mechanisms of α-adrenoceptor-mediated positive inotropy in mouse ventricular myocardium. The sympathetic nervous system regulates acute and chronic control of myocardial growth and inotropy. The group focuses on ion channels involved in α-adrenoceptor-mediated positive inotropy in the neonatal mouse ventricle. Through β-adrenergic receptor stimulation noradrenaline generates positive inotropy and chronotropy in most mammalian species, the effect on α-adrenoceptor stimulation is species- and time-dependent. During the postnatal development of the mouse right ventricular myocardium, the α-adrenoceptor-mediated inotropy changes from positive to negative. By evaluating the effects of different antihypertensive drugs on isolated neonatal mouse ventricles, the authors suggest that α-adrenoceptor-mediated positive inotropy is mediated by an increase in Ca^2+^ influx through the L-type Ca^2+^ channel. Furthermore, α-adrenoceptor-mediated positive inotropy was accompanied by prolongation of action potential duration, which in turn enhanced α-adrenoceptor-mediated positive inotropy [7]. In this Special Issue, Rosemary F. Kelly’s group investigated the effects of placing an adjuvant mesenchymal stem cell patch during coronary artery bypass surgery (CABG) [8]. Intramyocardial injection of mesenchymal stem cells has already been shown to improve left ventricular (LV) function in patients with chronic ischemic cardiomyopathy undergoing CABG by reducing fibrosis, neoangiogenesis, and neomyogenesis [9]. However, the group around R.F. Kelly aimed at investigating in more detail the effects of mesenchymal stem cell therapy in the disease setting of hibernating myocardium (HIB). HIB refers to the presence of persistently impaired LV function at rest, due to a reduced coronary blood flow that can be partially or completely restored to normal after revascularization. Using a porcine model of HIB, they found that diastolic function in HIB remained impaired despite revascularization with CABG. The authors further investigated the potential beneficial effects of proliferator-activated receptor-gamma coactivator PGC-1α upregulation induced by stem cell therapy. Indeed, adjuvant stem cell therapy during revascularization decreased pro-inflammatory cytokine expression and increased PGC-1α mediated mitochondrial biogenesis, resulting in an improvement of diastolic function [8].

Mei-Ling Cheng et al. elucidated in their contribution to this Special Issue the effects of 7-Ketocholesterol (7KCh), which results from oxidation of cholesterol and can be frequently detected in vascular atherosclerosis plaques and in the plasma of patients at high risk of cardiovascular diseases. Using cardiomyocyte cell lines, the authors demonstrate reduction in cardiomyocyte proliferation and an increase in mitochondrial oxygen consumption, accompanied by mitochondrial dysfunction and enhanced reactive oxygen species (ROS) production. Cardiac cells exhibited a compensatory increase in mitochondrial mass and an adaptative metabolic remodelling, characterized by a reduction in fatty acid oxidation (FAO). Reduced FAO was due to the accumulation of malonyl-CoA in 7KCh treated cardiomyocytes. Malonyl-CoA inhibits CPT-1, which is involved in the fatty acid uptake into the mitochondria and β-oxidation. The accumulation of malonyl-CoA might therefore represent a cardiac cell protective mechanism upon 7KCh treatment to reduce the use of fatty acids as fuel molecules for β-oxidation [10]. The group around Josep Julve deciphers in part the molecular mechanism by which FTY720, an FDA-approved sphingosine derivative, improves mitochondrial function in cardiomyocytes. Initially used for the treatment of multiple sclerosis, FTY720 has also been demonstrated to improve metabolic diseases. Using a human cardiomyocyte cell line, the authors demonstrate that FTY720 activates STAT3 which in turn stimulates mitochondrial activation. STAT3 inhibitor treatment reverses this effect, indicating specificity in the beneficial consequences of FTY720 activation on cardiomyocyte mitochondrial function through STAT3 activation [11]. Ariana P. Vargas-Delgado and co-authors discuss in their review for this Special Issue the metabolic impact of Sodium–glucose cotransporter type 2 inhibitors (SGLT2i) on the cardiovascular system. Originally developed for the treatment of type 2 diabetes mellitus, SGLT2is has been shown to be beneficial for renal and cardiovascular functions also in non-diabetic subjects. This review nicely summarizes the results of several clinical trials and details the effects of SGLT2is on kidney and cardiovascular functions [12]. Similarly, Peroxisome Proliferator Activated Receptor (PPAR) modulators are known to represent potent regulators of energy homeostasis and were initially designed to treat metabolic disorders such as diabetes mellitus and hyperlipidemia [13]. In our review, we summarized the main cardiovascular actions of PPAR modulators and the knowledge about their effects on the cardiovascular system and their safety in the treatment of cardiovascular diseases, based on the results of clinical trials. We furthermore put conventional cardiovascular therapies in context to PPAR related mechanisms of action. Based on the outcome of clinical trials, the use of PPAR modulators in cardiovascular diseases appears limited. However, several well established treatments such as Aspirin, Statins, Angiotensin-converting enzyme (ACE) inhibitors, and Angiotensin receptor blockers (ARBs) partially rely in their action on PPAR mediated mechanisms [14]. 

Very nicely, the group around Jean Armengaud demonstrates the power of combining modern molecular and imaging approaches to analyze historical samples in order to advance in our anthropological understanding of human history. They analyzed the reliques of the heart of a french saint and missionary figure born in 1799 using metaproteomics and high-resolution microtomography imaging approaches. Although historical narrations pointed to a death due to cardiovascular disease, the scientists could not find any evidence for a cardiac illness using these modern techniques and conclude death of this saint mostly to be due to tuberculotic and/or fungal pulmonary infection [15]. 

Using direct-infusion high-resolution mass spectrometry, the group of Annette F. Baas performed a study to comprehensively compare metabolites among severely and mildly affected carriers of Hypertrophic Cardiomyopathy (HCM)-causing variants. They could associate several metabolic pathways, including histidine, lysine, acylcarnitine, purine and steroid hormone metabolism with cases of severe HCM, which might in the future give additional indications for the pathogenesis, prognostic, and therapeutic care of patients with HCM [16]. Francesca Di Lorenzo and colleagues propose to define variants in the Desmoplakin (DSP) gene as an own clinical entity in the case of arrhythmogenic cardiomyopathy (ACM) and dilated cardiomyopathy (DCM). They analyzed 18 subjects, characterized as heterozygotes for DSP variants employing a target Next Generation Sequencing (NGS) cardiomyopathy panel. All patients exhibited a delayed enhancement (DE) area consistent with left ventricular (LV) myocardial fibrosis. Although variants in the DSP gene have been associated with both DCM and ACM phenotypes, some DSP case series reported in patients the occurrence of recurrent myocarditis, potentially representing the initial manifestation of cardiomyopathy, preceding systolic dysfunction. This study adds to the notion that DSP-related cardiomyopathy could be regarded as a distinct clinical entity from classic right ventricular arrhythmogenic cardiomyopathy (ARVC) and DCM, characterized by a fibrotic and inflammatory component, a high arrhythmic burden, and variable degrees of left ventricular enlargement [17]. A review from Igor Diembergers group for this Special Issue of IJMS focuses on atrial natriuretic peptide (ANP), a hormone secreted by the cardiac muscle cells of the atria in response to stretching of the atrial wall due to increased blood volume. ANP causes a reduction in expanded extracellular fluid volume by increasing renal sodium excretion. The authors review the diagnostic and therapeutic relevance of ANP in cardiovascular disease, especially atrial fibrillation and heart failure and discuss possible repercussions of cardioversion procedures and cardiac chirurgical interventions on ANP levels [18]. In his review for this Special Issue, Ivan Melnikov and co-authors analyze the knowledge about the impact of the NLRP3 inflammasome, especially the different C-reactive protein (CRP) isoforms, on atherosclerotic cardiovascular disease. They further discuss the consequences of established anti-inflammatory therapies in the context of atherosclerotic cardiovascular disease [19]. Given the worldwide success of COVID-19 vaccination, with is mainly RNA based, potential RNA therapeutics became a very hot topic in the scientific community worldwide. Modified mRNA (modRNA) applications in cardiology are also progressing quickly, and Ajit Magadum provides in his review for this Special Issue an overview about the methodology required, the potential therapeutic applications, and finally discusses the need of approaches to develop efficient mRNA delivery systems that can ensure targeted, non-invasive gene delivery into the heart [20].

This Special Issue “Molecular Mechanisms of Cardiac Development and Disease” represents a valuable collection of studies that aim at elucidating molecular mechanisms in cardiac development, disease, repair, and regeneration. Several studies describe recent advances and perspectives in the diagnostics and treatment of cardiovascular diseases, which contributes to our understanding of cardiac disease and hopefully accelerates the development of novel, effective therapies. 
